# Gastrostomy tube dislodgment acute pancreatitis

**DOI:** 10.1186/1749-7922-9-23

**Published:** 2014-03-28

**Authors:** Eran Brauner, Yoram Kluger

**Affiliations:** 1 Department of General Surgery, Rambam Health Care Campus, Haifa, Israel

**Keywords:** Pancreatitis, Gastrostomy, Percutaneous endoscopic gastrostomy (PEG), Foley catheter

## Abstract

Percutaneous gastrostomy is well established root for long term feeding of patients who cannot be fed orally. The risks of percutanous gastrostomy insertion are low. Tube related complications often resolved by placing a Foley catheter or other balloon gastrostomy tube as a temporary solution. Gastrostomy tube related gastric, duodenal and billiary obstruction were reported. Gastrostomy tube related pancreatitis is scarcely described. We described a patient who suffered a pancreatitis related to Foley catheter gastrostomy dislodgment. Reviewing all reported cases of gastrostomy related pancreatitis revealed higher incidence in patient with Foley catheter used as gastrostomy and revealed questionable trends in conducting tube replacement. We suggest a proper manner for tube replacement and concluded that should a Foley catheter used as a temporary solution a replacement should be schedule in a timely manner to avoid life threatening complications.

## Introduction

Percutaneous gastrostomy is the preferred root for long term feeding of patients who cannot be fed orally [1]. The use of percutaneous gastrostomy carries a low risk for complications. Listed among the potential life threatening complications of this procedure is obstructive pancreatitis resulting from migration of the tube and obstruction of the 2^nd^ part of the duodenum by the catheter's balloon. This complication is rare and only scarcely described in the English literature. Usually, when a tube related complications are encountered a Foley catheter is placed instead of a designated tube. Therefore physician taking care of patients feed via feeding tube should be aware of this complication.

Herein we describe a patient who presented to the emergency department with abdominal pain. Eventually he was diagnosed with pancreatitis resulting from the Foley catheter migration in to the 2^nd^ part of the duodenum. We review all published cases of pancreatitis related to feeding tube migration and suggest safety manner for tube replacement.

## Case presentation

A ninety two year old patient, a resident of a nursing home, presented to the emergency department with acute general deterioration and coffee ground vomiting.

Her medical history consisted with Alzheimer's dementia and CVA (cerebro vascular accident) that resulted in dysphagia.

The patient had a percutaneous endoscopic gastrostomy (PEG) tube inserted two years prior to her admission. The PEG was replaced with a Foley catheter a year ago due to inadvertent dislodgment while nursing the patient.

At presentation the patient was agitated. Her blood pressure was 90/60 mmHg. Her oxygen saturation was 90%. Physical examination revealed a tender abdomen. The gastrostomy tube drained coffee ground material. Laboratory studies showed marked leukocytosis of 23000 and Creatinine level was 1.4 mg/dl. Urinalysis showed amylase level of 11,460 U/L.

Plain abdominal and chest radiograph were normal. No free air was detected.

An upper abdominal Ultrasound was preformed, demonstrating an enlarged gallbladder with no gallstones or sludge. There were no signs of cholecystitis but the common bile duct (CBD) was dilated to 16 mm.

**Figure 1:**
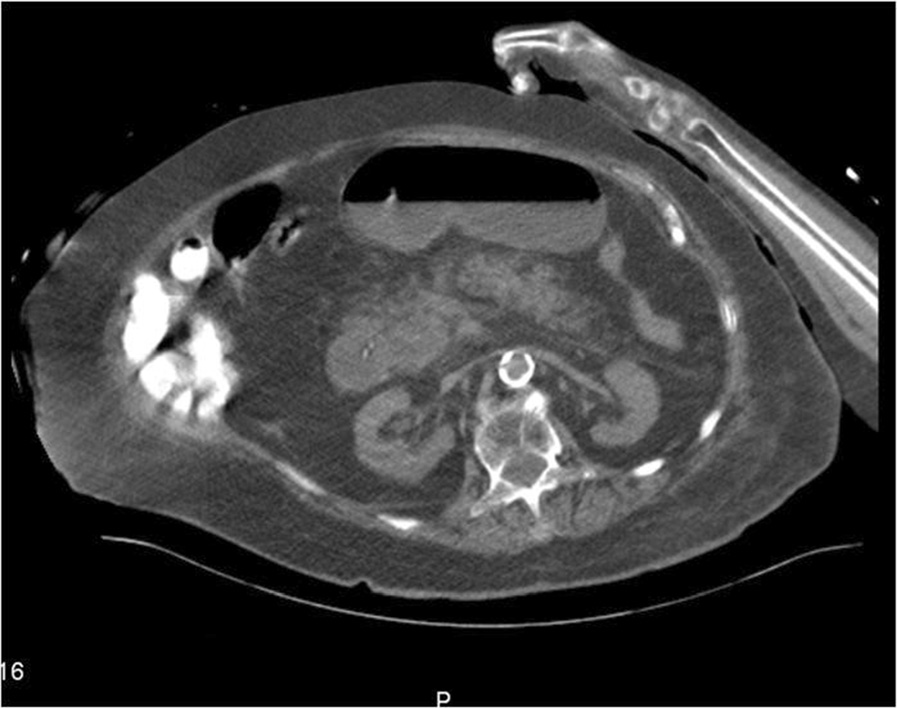
Abdominal CT scan showing Foley catheter balloon located in the second part of the duodenum and peripancreatic fat stranding with an edematous pancreatic head.

An abdominal CT with IV contrast revealed a peripancreatic fat stranding and an edematous pancreatic head. These finding were consistent with acute pancreatitis. The Foley catheter balloon was seen deep in the second part of the duodenum facing Vaters' papilla (Figure 1).

The gastrostomy tube was pulled back to the stomach and secured to the abdominal wall with silk stich. The patient was treated with fluid and analgesics. The next day a follow-up sonographic evaluation was done indicating a reduction of the CBD diameter to 11 mm.

During her stay in the hospital her respiratory symptoms were significantly relieved, she regained hemodynamic stability, was normothermic and her abdominal tenderness disappeared. Laboratory results normalized. Bilirubin and amylase levels returned to normal within three days of her admission.

She was discharged after 6 days, having significantly improved and was sent back to her retirement home.

## Discussion

Percutaneous Endoscopic Gastrostomy (PEG) tube was first described in 1980 by Gaunderer [2]. PEG is consider safe and effective method for providing long term enteral nutrition while offering advantages over nasogastric tube feeding [3,4].

The incidence of short and long term complications related to PEG actual insertion is low [5]. However, tube related complications such as granulation tissue, broken or leaking tube, leakage around the tube site and stomal site infection exceed 60% [6]. Migration of feeding gastrostomy has been described in the past as the cause for gastric outlet obstruction [7], duodenal obstruction [8] and biliary obstruction [9].

Our case presents pancreatitis as a potential complication of a balloon gastrostomy tube. In our case it seems that the Foley catheter's balloon obstructed the ampulla of Vater, therefore resulting in acute pancreatitis.

Gastrostomy tube dislodgement pancreatitis is rare. Review of the English literature revealed 10 cases of pancreatitis as a result of migration of feeding gastrostomy [5,10-17]. The first case was published in 1986 by Bui et al. [10]. He described a migration of a Foley catheter that was inadvertently left in place after establishing a permanent surgical Gastrostomy.

Selected characteristics of all eleven cases, (including our patient) are outline in Table 1. Reasons for gastrostomy tube placement varied with age, from mental retardation and cerebral palsy in the younger age to CVA in older patients. Time from the replacement of the tube to initiation of symptoms varied widely from one day to one year. None of the published cases described this complication with a new inserted PEG. In all cases, balloon feeding tube was used as a temporary solution in a well and established tract.

**Table 1:** Characteristics of cases of feeding tube dislodgment pancreatitis

**Ref no.**	***Age (y)***	***Gender***	***Type of catheter***	***Diagnosis***	***Time from replacement to presentation***	***Replacement set-up***	***Repositioning confirmation test***
10	37	m	Foley	Barium study	1 day	NM	None
11	11	m	Foley	Barium study	1 day	Home	None
12	32	f	Foley	Incidentally by ERCP	6 month	Medical facility	EGD
13	26	f	Balloon gastrostomy w/external disk bumper	CT	3 month	NM	NM
14	44	m	Foley	ECRP	NM	NM	NM
15	57	f	Balloon gastrostomy w/external disk bumper	MRCP	4 weeks	NM	NM
16	86	f	Balloon gastrostomy w/external disk bumper	CT	4 weeks	Home	None
17	25	f	PEG w/ external disk bumper	CT	3 days	Home	None
5	79	m	Foley	CT	Few days	Home	None
5	38	f	PEG w/ external disk bumper	CT	NM	NM	NM
-	92	f	Foley	CT	1 year	Home	None

NM- not mentioned, ERCP- endoscopic retrograde cholangiopancreaticography, EGD- esophago gastroduadenoscopy, CT- computed tomography, MRCP- magnetic resonance cholangiopancreaticograohy, PEG- percutaneous endoscopic gastrostomy.

One case [12] describes the insertion setup to be in a medical facility and its position was confirmed using upper endoscopy. In all remaining cases the insertion setup was not mentioned (5 cases) or was at the patient's bedside (5 cases). In most instances (54.5%) no active test was done to confirm the new feeding tube position.

Tube related complication is often managed by replacing the PEG with a Foley catheter as a bridging solution, in the acute setting at the emergency room or the patient's bed side in nursing homes. In six of the reported cases (54.5%) Foley catheter was used and five (45.5%) reported the use of a balloon gastrostomy tube with external bolster.

One of the major disadvantages of the Foley catheter at this non formal but common use is the lack of a stopper mechanism which prevents the catheter from propelling distally with peristalsis.

Our case strengths the assumption made before [5] that the use of Foley catheter as a gastrostomy tube increases the risk of pancreatitis and should be avoided. Nevertheless in case of a Foley catheter is used as a bridging solution for a mechanically failed formal gastrostomy tube, early definitive proper elective replacement of the Foley catheter should be practiced in order to avoid potentially life threatening conditions. We strongly recommend replacing the failed or broken original feeding tube in a medical facility in order to confirm its position radiographically before using the tube. Marking the catheter at its skin level can help tracking its position periodically. We suggest that whenever a patient with feeding gastrostomy is diagnosed with pancreatitis or obstructive jaundice its position should be identified using contrast material injected through the tube. And should the diagnosis of tube dislodgment pancreatitis is made, deflating the catheter balloon and withdrawing the tube can reverse all pathologic laboratory findings and may result in the patient's prompt recovery.

## Consent

Written informed consent was obtained from the patient's daughter for publication of this Case report and any accompanying images. A copy of the written consent is available for review by the Editor-in-Chief of this journal.

## Competing interests

All authors declare that they have no competing interests.

## Authors’ contributions

EB conceived of the study, performed the literature search and carried out the drafting of the manuscript. YK participated in coordination and helped to draft the manuscript. All authors read and approved the final manuscript.
